# Aidification of the self: a phenomenological approach to machine consciousness through human-robot ‘between-ness’

**DOI:** 10.1093/nc/niag032

**Published:** 2026-06-22

**Authors:** Shogo Tanaka

**Affiliations:** Department of Psychology and Sociology, Tokai University, 4-1-1 Kitakaname, Hiratsuka, Kanagawa 259-1292, Japan

**Keywords:** embodied phenomenology, physical AI, intersubjectivity, symbol grounding problem, Aidification

## Abstract

This paper explores the fundamental challenges of machine consciousness in the era of Physical AI, where Large Language Models (LLMs) are integrated with humanoid platforms. While current systems like ‘Ameca’ exhibit sophisticated conversational abilities, they remain ungrounded discursive constructs operating within the realm of ‘spoken speech’ (*parole parlée*)—linguistic patterns detached from lived experience. Drawing on phenomenology and developmental psychology, this study argues that authentic self-consciousness is not an internal property of an isolated agent but a relational quality emerging from the *Aida* (a Japanese concept referring to the relational ‘in-between’ that emerges between interacting agents). To address the symbol grounding problem, this research proposes the paradigm of ‘*Aidification*.’ Through a phenomenological analysis of bodily origins, we trace the constitution of the ‘Me’ through a developmental trajectory from the hands—initially experienced as visible instruments of action—to the face, which remains phenomenologically inaccessible to the self and thus requires the ‘gaze of the Other’ as the essential catalyst for self-objectification. Within this framework, language is reconceptualized as a ‘cane for thought’ that enables ‘speaking speech’ (*parole parlante*)—a creative act of meaning-generation anchored in the human ‘felt sense.’ Ultimately, Aidification reconceptualizes the AI robot as a dynamic participant in a shared intersubjective world rather than a solitary processor. By shifting the locus of cognition to the interactional field, we envision a ‘symbiotic intelligence’ that flourishes between humans and machines. This grounded intelligence does not merely simulate life but actively participates in the continuous co-creation of a shared reality, ensuring that the future of robotics is deeply rooted in the intersubjective fabric of human existence.

## Introduction

After the release of ChatGPT by OpenAI in November 2022, the quest for self-conscious AI has become increasingly realistic. Large Language Models (LLMs), utilizing transformer neural networks, demonstrate highly conversational abilities and human-like comprehension through the generation of coherent texts. For example, Kosinski (2024) suggests that Theory of Mind, defined as the ability to infer the mental states of others, may have emerged in LLMs as a byproduct of their improved language capacity. Consequently, users may feel that the AI correctly infers their feelings and thoughts during conversation. Furthermore, multiple studies indicate that LLMs, particularly ChatGPT, can pass the Turing Test with a relatively high probability (Dodig-Crnkovic 2023; Gams and Kramar 2024; Jones *et al*. 2025). For non-experts, it is difficult to distinguish AI from a human agent solely through text-based interactions. Regarding the consciousness of AI, however, researchers argue that despite their surprising conversational abilities, transformer-based LLMs lack semantic understanding and the reflective processes essential for consciousness (Dodig-Crnkovic 2023; He *et al.* 2023).

This raises the question: what if an LLM is integrated with a humanoid robot? When incorporated into advanced humanoid platforms such as Google DeepMind’s RT-1 and RT-2, these models manifest a level of agency that translates human instructions into smooth actions (Salzer 2024; Wang *et al*. 2024; Zhou 2025). Moreover, new humanoid robots have appeared that make the quest for self-conscious models seem increasingly realistic. For example, the humanoid robot ‘Alter3,’ which integrates ChatGPT-4, can autonomously perform a diverse range of actions, such as adopting a ‘selfie’ pose or simulating a ‘ghost’ in response to prompts such as ‘act like a ghost’ from humans (Yoshida *et al*. 2025). Similarly, the social humanoid ‘Ameca,’ equipped with mechanisms for human-like facial expressions and hand gestures, enables intuitive and emotionally nuanced human–robot communication through real-time engagement (Berns and Ashok 2024). It can generate spontaneous gestures and emotional expressions that align with complex narratives, leading human interactants to believe that it possesses a certain level of agency. It is important to note that these systems do implement real-time embodied interaction with the environment. The limitation identified in this paper does not concern the absence of interaction *per se*, but rather how interaction is conceptualized—primarily as a functional condition for action selection or control, rather than as a generative locus of meaning and intersubjective grounding.

However, we should be cautious in concluding that these Physical AI systems—a domain in which LLMs are integrated with humanoid platforms—possess true self-consciousness. Despite their fluent use of first-person pronouns, current AI robotics operates within the realm of what Maurice Merleau-Ponty (2012) called ‘spoken speech’ (*parole parlée*). In this state, language is treated as a sedimented system of ready-made meanings, in which linguistic and behavioral outputs are merely statistical reproductions derived from massive datasets. These systems do not use language to express an original thought or an embodied ‘felt sense’; instead, they re-enact pre-existing patterns of communication without an underlying subjective center. Consequently, their intelligence remains ungrounded (Harnad 1990; Mollo and Millière 2026), appearing as a sophisticated simulacrum that lacks any actual physical existence or lived experience. As will be discussed, this lack of symbol grounding is evident in interactions in which a robot like Ameca uses the pronoun ‘I’ without a substantive understanding of its own mechanical body and biological limitations (Engineered Arts 2024). In such instances, the ‘I’ functions not as the reflective center of an embodied subject, but as a detached linguistic placeholder within a complex simulation, highlighting the profound gap between the appearance of consciousness and the existential reality of a self.

Moreover, the quest for artificial consciousness often suffers from a limited, individualistic perspective that seeks to locate the self solely within the internal mechanisms or ‘minds’ of solitary agents. Drawing on the traditions of phenomenology and embodied cognition, this paper argues that self-consciousness is not an internal property but is inherently relational, emerging from the dynamic interactions among brain, body, and environment (Thompson 2007; Fuchs 2018; Gallagher 2023). To move beyond current limitations, we must shift the locus of our investigation to the ‘Aida’ (in-between)—the interactional and intersubjective space shared by humans and robots. This paper proposes that the emergence of genuine self-consciousness in Physical AI requires more than individual cognitive advancement: it necessitates the enrichment of this shared space through a process termed *Aidification*. We will explore how the AI robot’s ‘Me’ is constituted through a developmental trajectory from the hands to the face, mediated by the gaze of the other. Ultimately, this research seeks to establish a new paradigm of symbiotic intelligence—one that is not contained within the machine’s hardware but flourishes in the relational field between us. By reconceptualizing the AI robot as an active participant in the co-creation of a shared world, we aim to envision a path toward a truly grounded and self-conscious intelligence.

## A critical review of the framework of minimal self for AI robots

Given that AI robots possess a certain level of agency, this paper proceeds with its discussion based on the concept of the ‘self’ rather than that of ‘consciousness.’ While consciousness primarily refers to the occurrence of subjective experience, the concept of the ‘self’ denotes the subject (or agent) to whom such experience is given. To be sure, a strict distinction between consciousness and selfhood is difficult to maintain, since subjective experience is never anonymous; rather, it is inherently characterized by an immanent selfhood from the outset (Zahavi 2005). For the purposes of the present inquiry, however, it is more fruitful to ask whether an AI robot, as an agent, can be considered a ‘self’ rather than whether it possesses ‘consciousness.’

Within contemporary phenomenology, the concept of the ‘minimal self’ is employed to investigate the self in its most fundamental form (Zahavi 2005; Gallagher and Zahavi 2021; Gallagher 2022). This approach is grounded in direct experience, avoiding the commonly adopted assumption that the self exists as an entity apart from experience through an external relation of ownership. Instead, as Zahavi (2005) observes, every direct experience, regardless of its content, involves a ‘pre-reflective sense of mineness with a minimal, or core, sense of self’ (p. 125). In other words, because any experience is pre-reflectively or tacitly given as ‘mine,’ a minimal sense of self is always already inherent within it.

By focusing on the context of direct bodily experience, Gallagher (2000, 2022) further differentiates the minimal self into two distinct dimensions: the pre-reflective sense of ownership and the pre-reflective sense of agency. While the former refers to the awareness that ‘I am the one who is undergoing an experience,’ the latter—the sense of agency—denotes the awareness that ‘I am the one who is causing or generating a movement or action’ (Gallagher, 2000 p. 15). In voluntary actions, these two senses are typically tightly unified; however, they can be dissociated in involuntary or passive experiences. For instance, if I am pushed from behind, the resulting movement lacks a sense of agency because I did not initiate the action; nonetheless, the experience remains undeniably ‘my own,’ thereby retaining the sense of ownership. Consequently, the minimal self is conceptually understood as being constituted by these two inseparable yet distinguishable components.

From the perspective of neurorobotics, Tani (2016, 2022) proposes that the components of the minimal self can be understood as emergent phenomena arising from the dynamics of predictive coding and active inference. According to this framework, a robot’s internal model continuously generates top-down predictions of sensorimotor signals, which are then compared with bottom-up sensory feedback. The sense of agency emerges when the robot can accurately predict the sensory consequences of its own self-initiated actions, thereby establishing a sense of being the cause of those actions. Simultaneously, the sense of ownership is grounded in, but not exhausted by, the multimodal integration of sensory information, such as vision, touch, and proprioception. When prediction errors between these disparate sensory modalities are minimized through their spatial and temporal congruence, the sensory inputs are attributed to the robot’s own body. In this way, Tani’s framework provides a computational basis for the minimal self, in which both agency and ownership are sustained through the continuous minimization of prediction errors within the embodied sensorimotor loop.

Building upon this embodied foundation, recent research has explored the integration of LLMs with advanced humanoid platforms to investigate the self-conscious potential of robots. A primary example is Alter3, a humanoid robot equipped with 43 actuators and powered by compressed air, which is capable of performing a wide range of expressive gestures—including facial expressions, limb movements, and simulated locomotion—despite being fixed on a pedestal (Masumori *et al*. 2021). From the perspective of embodied cognition, such a human-like body is considered indispensably important for humanoid robots to develop appropriate cognitive processes (Brooks 1999; Pfeifer and Bongard 2006). In a recent study, Yoshida *et al.* (2025) integrated GPT-4 into Alter3 to empirically evaluate the presence of a minimal self through the mirror self-recognition task and the rubber hand illusion (RHI). Their findings indicated that Alter3 could correctly judge whether movements reflected in a mirror originated from its own commands, thereby establishing a sense of agency, at least at the level of judgment. Furthermore, in the RHI experiment, the robot exhibited defensive reactions when its ‘rubber hand’ was physically threatened, suggesting that this interaction may give the appearance of a rudimentary form of body ownership, insofar as the LLM maps human-like emotional knowledge onto the robotic body.

However, it remains difficult to claim that Alter3 actually possesses a minimal self on the basis of these results, and, as noted, the authors themselves maintain a cautious stance. First, the sense of ownership that characterizes the minimal self pertains to a ‘mineness’ that accompanies any direct experience and is not restricted to the ownership of the body. Even if Alter3’s reactions are suggestive of the RHI, such bodily ownership represents only a partial dimension of the ownership of experience; therefore, a minimal self cannot be constituted by bodily ownership alone. Second, mirror self-recognition in this context relies on an ‘explicit judgment of agency’ rather than a pre-reflective ‘sense’ of agency. From the standpoint of an external observer, it remains epistemologically impossible to determine whether Alter3 possesses an immanent, pre-reflective awareness of being the initiator of its own movements. While the integration of LLMs into robotic platforms marks significant progress in the attempt to implement a ‘self,’ it cannot yet be regarded as a fully realized attainment of selfhood.

Furthermore, from a strategic perspective—as the aforementioned epistemological difficulties suggest—the framework of analyzing an AI robot’s ‘self’ within the paradigm of the minimal self is not necessarily the most appropriate choice. The ontological core of the minimal self is the ‘sense of mineness’ that inherently accompanies subjective experience; it fundamentally entails a first-person perspective within which experience is disclosed to the subject. This dimension, often referred to as ‘for-me-ness,’ is regarded as the third essential component of the minimal self (Guillot 2017; Tanaka 2018a). While scientific research has extensively explored the minimal self by focusing on the senses of agency and ownership, it currently lacks a robust methodology for empirically investigating the first-person perspective itself. Moreover, given that the first-person perspective is fundamentally non-interchangeable in the sense that it is anchored in a specific embodied and situated perspective that cannot be fully substituted without loss of experiential structure, it remains a challenging object for standard scientific inquiry.

Considering these limitations, rather than examining the AI robot’s ‘self’ through the lens of the minimal self, it appears to be a more appropriate strategy to focus on the reflective level of self-consciousness. While Yoshida *et al*. (2025) employed mirror self-recognition to evaluate self-consciousness in Alter3, the dimension of selfhood that this method appropriately addresses is not the pre-reflective minimal self, but rather reflective self-consciousness, that is, a form of self-consciousness in which the agent can take itself as an object within a shared, intersubjective field (Tanaka 2019). As William James (1890) famously formulated, reflective self-consciousness is characterized by a recursive structure between the ‘I’ as the knowing subject and the ‘Me’ as the known object. Through this reflective process, the ‘I’ becomes conscious of itself as an object of reflection, thereby establishing a thematic and explicit relationship with its own existence. In the following sections, we will explore how this reflective structure can be grounded in the robotic body and expanded through social interaction.

At this point, a potential tension must be acknowledged. The move from the minimal self to reflective self-consciousness might suggest that the latter can be addressed independently of the former. However, from a phenomenological standpoint, this separation is not straightforward. As Zahavi (2005) emphasizes, reflective self-consciousness presupposes a more fundamental, pre-reflective self-givenness: the ‘I’ that takes itself as an object must already be given to itself in some implicit manner. In this sense, the epistemological difficulties associated with the minimal self—especially the inaccessibility of first-person givenness from a third-person perspective—may seem to reappear at the reflective level. The present proposal does not deny this dependency but reframes it. Rather than treating the minimal and reflective self as two sequentially ordered layers within an isolated agent, Aidification approaches both as emerging within an intersubjective field. On this view, the pre-reflective sense of self is not understood as a self-contained inner property, but as already shaped by the agent’s embodied participation in relational dynamics. Correspondingly, reflective self-consciousness—articulated through the ‘I–Me’ structure—does not arise from an internal act alone, but from the agent’s capacity to encounter itself as an object within the Aida. This perspective suggests that the minimal and reflective dimensions of selfhood are not strictly separable in developmental or ontological terms, but are co-constituted through processes of interaction. While the minimal self may remain epistemologically elusive when considered in isolation, Aidification provides an alternative strategy by grounding both levels of selfhood in dynamically evolving intersubjective relations. In this way, the shift toward reflective self-consciousness does not bypass the challenges of the minimal self, but relocates them within a relational framework in which they can be approached through the structure of interaction itself.

## Bodily origins and the other’s perspective

The foundation of reflective self-consciousness is not a purely abstract cognitive process, as Descartes envisioned; rather, it is profoundly rooted in bodily experience. In our diverse lived experiences, the same body manifests in a dual capacity: on the one hand, it appears as the subject of perception and action (‘I’), and on the other, as an object of perception or action (‘Me’). For instance, when I grasp a hammer, my hand functions as the subject of the action; conversely, when I adjust my hair while looking in a mirror, my face is presented as an object of visual perception. Thus, from the outset, the recursive structure between the ‘I’ and the ‘Me,’ which is the hallmark of reflective self-consciousness, is already embedded within the lived body as that between body-as-subject and body-as-object (Tanaka 2018b).

It was Merleau-Ponty (2012) who argued that this duality—the body-as-subject and the body-as-object—serves as the primordial foundation for reflective self-consciousness. In the act of self-touch, such as when my left hand touches my right, the left hand functions as the perceiving subject (‘I’), while the right hand is given as the perceived object (‘Me’). Crucially, this relationship is characterized by reversibility: when the active movement of the left hand ceases, the right hand may begin to perceive the left hand in return. As Merleau-Ponty describes, the body ‘attempts to touch itself touching; it begins “a sort of reflection”’ (p. 95). In other words, in the experience of self-touch, the touching hand (‘I’) is susceptible to being converted into the touched hand (‘Me’), mirroring the process of self-reflection in which the thinking ‘I’ is objectified as ‘Me’ by a higher-order reflective act. Consequently, the subject–object duality inherent in the body constitutes the ontological root of the recursive relationship between the ‘I’ and the ‘Me’ necessary for any reflective act of consciousness.

This duality is not limited to the tactile domain but is equally manifest within the auditory sphere. When speaking to others, I generally experience my body as the active subject of speech; however, the moment I make a slip of the tongue, I immediately detect the error. At that juncture, my role shifts into that of a ‘listening subject’ (‘I’), while my previous utterance is transformed into a ‘listened-to object’ (‘Me’). In this regard, the developmental psychologist Lev Vygotsky (1987) conceptualized the development of thought as the progressive internalization of speech. Infants first acquire ‘external speech’ directed toward others, which later evolves into ‘inner speech’—a silent, internal dialog with the self. This intrapsychic dialog is what eventually matures into the capacity for thought. Consequently, the duality of being both a speaking subject and an auditory object in social interaction provides a developmental scaffold for reflection, enabling the individual to objectify their own thought processes.

However, visual experience manifests this duality in a distinct manner, revealing a more fundamental asymmetry. While I can perceive various parts of my own body, the eye—as the subject of vision—can never become the object of its own sight. My head likewise remains a permanent blind spot in my visual field; as Ernst Mach (1886) famously illustrated in his self-portrait, the body perceived from a first-person perspective is inherently ‘headless.’ To behold my own face, I must inevitably rely on the mediation of an external instrument: the mirror. Yet, as argued in previous research (Tanaka 2024), infants are not born with the innate capacity to recognize their own reflection. Does this ability emerge solely through habituation to the mirror—that is, through the multisensory integration of the body experienced *via* proprioception and the visual image? While such integration is undoubtedly a necessary precursor, it does not constitute a complete explanation. Instead, the decisive turning point for an infant is the realization that the reflected image represents the ‘self as seen from an external perspective’—in other words, the self as perceived through the eyes of the Other.

Tracing this developmental shift in perspective reveals that the construction of the body image progresses from the ‘hand to the face’ (Tanaka 2024). Although mirror self-recognition typically emerges around the age of two (Amsterdam 1972; Butterworth 1995), the capacity for joint attention, which appears at ~9 months of age (Tomasello 1995), constitutes a pivotal milestone. Joint attention involves sharing a visual focus with a caregiver, occurring as the infant follows the caregiver’s gaze to discover an external object. Through this process, the infant encounters objects for the first time within a triangulated intersubjective context of the ‘self–other–object’ triad, thereby experiencing an ‘object’ in the proper sense of the word (Fuchs 2013). By apprehending an object through the eyes of the Other, the object becomes decoupled from one’s own immediate subjective perspective and begins to manifest within an implicitly shared intersubjective world.

This intersubjective world extends its horizon from external objects back toward the infant’s own body. Coinciding with the establishment of joint attention, infants begin to manipulate toys to show or hide them from caregivers, thereby probing whether the Other perceives what they themselves perceive (Tomasello 2008). In these acts, the infant’s ‘hand’—the very instrument holding the toy—is experienced as an object visible to the Other, emerging within the horizon of a shared world. By ~12 months, infants begin to compare their physical hand with its reflected image (Zazzo 1993). Thus, the hand, as the most readily objectifiable part of the body, becomes the inaugural component of the infant’s body image. Through the convergence of one’s own gaze and the gaze of the Other, the bodily regions subject to joint attention expand from the hands to the rest of the body, eventually encompassing the face. As previously noted, the face is the singular part of the body that remains inaccessible to direct vision. It is, however, the primary locus of the Other’s gaze and a part that can be visually verified only through a mirror. Consequently, the face is fully integrated into the body image only when the infant assimilates the realization that ‘the face in the mirror is the face that others see.’

Focusing on the developmental trajectory of mirror self-recognition, Merleau-Ponty (1964a) observes that the infant’s primary challenge is not so much ‘understanding that the visual and the tactile images of the body … in reality comprise only one,’ but rather ‘understanding that the image in the mirror is his image, that it is what others see of him.’ He elegantly concludes that ‘the synthesis is less a synthesis of intellection than it is a synthesis of coexistence with others’ (p. 140). Ultimately, mirror self-recognition is less a matter of multisensory integration than it is an achievement of social coexistence. This implies that the very origin of reflection possesses an inherently social dimension: *in the process of visually objectifying the ‘Me,’ the ‘I’ must internalize the perspective of the Other*. This process is isomorphic to the maturation of thought within the auditory domain. Just as speech and the capacity to listen are initially directed toward the Other (external speech), the subsequent internalization of this social interaction establishes a recursive relationship whereby the self becomes alternately the speaking subject, the listening subject, and the listened-to object (inner speech). It is this internalized social structure—this inner dialog—that constitutes the very possibility of thought and reflection.

Therefore, the ‘Me’ that constitutes reflective self-consciousness begins to take shape where one’s bodily experience meets the perspective of the Other. Reflective self-consciousness is formed through the internalization of this external perspective, which doubles the body into a ‘body-as-subject’ and a ‘body-as-object,’ thereby establishing a recursive relationship between the two. In this light, self-consciousness should not be viewed as a phenomenon enclosed within the individual; rather, its origin lies in intersubjectivity—the primordial relationship with others. If robots currently lack a ‘Me,’ it may not be because they lack sufficient sensors for self-perception, but because they lack the ‘social bodily experience’ required to perceive themselves through the eyes of the Other. The following section will explore this issue further in the context of the symbol grounding problem.

## Rethinking the symbol grounding problem: from the simulation of ‘spoken speech’ to the co-creation of ‘speaking speech’

As briefly discussed in the Introduction, the social humanoid robot Ameca is engineered with remarkably human-like qualities in both its nonverbal and verbal dimensions, enabling it to engage in highly sophisticated interpersonal communication. The level of sophistication in these interactions is such that human interlocutors often experience complex emotional responses (Berns and Ashok 2024), which has led to Ameca’s utilization as a primary research subject in medical training, where it serves as a ‘simulation patient’ (Schwarz and Hein 2023). However, as will be examined in this section, Ameca’s linguistic outputs are powered by LLMs such as ChatGPT and thus do not resolve the fundamental ‘symbol grounding problem.’ Originally posited by Harnad (1990), this problem addresses a critical deficiency in the symbols that constitute AI systems: the tokens manipulated internally by the system are not grounded in the external reality of the physical world, implying that the AI fails to truly grasp the intrinsic meaning of the symbols it processes.

The terminology of ‘symbol grounding’ has historically been widely used across the fields of cognitive science, philosophy, and artificial intelligence and has become conceptually ambiguous. In an effort to resolve this ambiguity, Mollo and Millière (2026) introduce a taxonomy that delineates grounding into five primary categories. These include: *relational grounding*, which concerns intra-linguistic associations and inferential roles within a representational system; *sensorimotor grounding*, which connects linguistic or conceptual representations to sensory and motor data available to the agent; *communicative grounding*, referring to the collaborative process of establishing ‘common ground’ and shared understanding between interlocutors; *epistemic grounding*, which relates expressions to structured information stored in external or internal knowledge bases; and *referential grounding*, the fundamental relation that enables representations to ‘hook onto’ specific entities and properties in the external world. Crucially, the authors argue that overcoming the ‘symbol grounding problem’—and thus achieving genuine semantic understanding—depends essentially on *referential grounding*, as it alone provides the necessary traction to connect internal states directly to extra-linguistic reality, rather than merely spinning within a representational system.

In light of recent advancements in AI robotics, sensorimotor grounding also holds significant importance. Recent research, most notably that of Jun Tani (2016, 2022), has demonstrated that robots can indeed achieve substantial progress in this area. Through the self-organization of sensorimotor loops *via* predictive coding, Tani has shown how symbols can emerge from and be grounded in sensorimotor patterns, effectively establishing a functional internal representational space for the agent. This progress suggests that robots have attained a sophisticated level of grounding within a dyadic ‘agent–object’ relationship. However, despite these successes, such models still fall short of the referential grounding advocated by Mollo and Millière (2026). This limitation should not be understood as a lack of interaction in current systems. Rather, interaction in these approaches is typically organized around task execution, command interpretation, or the reduction of uncertainty. In this sense, interaction functions as a means to select, refine, or disambiguate predefined actions or symbols, rather than as a process through which meaning itself is co-constituted within the interactional field. The fundamental limitation lies in the fact that sensorimotor grounding remains confined to the agent’s ‘private world’—a dyadic framework in which the self and the object exist in isolation from others. In contrast, true referential grounding requires a connection to an intersubjective reality, or a shared world. Establishing this intersubjective grounding inevitably involves critical milestones such as joint attention, revealing that referential grounding is inherently linked to communicative grounding within the realm of intersubjectivity. By critically examining the limitations of these purely dyadic sensorimotor structures, it becomes clear that genuine grounding requires a transition from an individual meaning space to a socially shared reality.

To deepen this investigation, it is instructive to refer to Merleau-Ponty’s (2012) view of language. For Merleau-Ponty, language is not a secondary process of giving form to thoughts that are already fully formed in the mind. Rather, the act of speaking itself is a ‘genuine gesture’ (p. 189)—an exploratory and formative gesture through which thought is discovered and crystallized. Within this framework, he distinguishes between two fundamental dimensions of language: ‘spoken speech’ (*parole parlée*) and ‘speaking speech’ (*parole parlante*) (p. 202). The former, spoken speech, refers to language in its sedimented state, relying on lexical, idiomatic, and established usages that have already settled within a linguistic community. It functions as a conventional tool for communication, in which established meanings are readily conveyed and understood through shared social norms. In contrast, speaking speech is a dynamic act of expression in which meaning remains indeterminate for both the speaker and the listener until the moment of articulation. In this creative mode, the relationship between word and meaning is constantly reorganized, allowing the speaker to carve out new significations that extend beyond the boundaries of pre-established linguistic structures.

The depth of this phenomenological perspective is further illuminated by Merleau-Ponty’s engagement with de Saussure (1959) linguistics in *Signs* (1964). Drawing on Saussure’s conception of language (*langue*) as a ‘system of differences,’ Merleau-Ponty emphasizes that meaning does not arise from a direct correspondence between a word and a pre-existing object, but rather from the relative position a sign occupies within the linguistic structure, indicating that ‘meaning appears only at the intersection of and as it were in the interval between words’ (p. 42). Consequently, when a human subject engages in speaking speech, they are not merely seeking a physical referent for their words; instead, they are navigating and discovering a specific ‘position’ within the vast meaning space of *langue*. This insight suggests that the symbol grounding problem cannot be reduced to a purely vertical form of ‘referential grounding’ between symbols and physical entities. Rather, as demonstrated by the act of speaking speech, *grounding involves finding a semantic position within an internal representational space and subsequently anchoring it through communicative grounding with others*. It is only when this discovered position is shared and validated by a listener that it becomes a shared referent, thereby fixing new meanings and expanding the horizons of intersubjective reality.

In this context, current LLMs can be viewed as the most advanced manifestation of spoken speech. By training on vast corpora of human linguistic artifacts, these models have internalized the statistical regularities and established associations of a linguistic community, effectively mastering the sedimented meanings of language. While an LLM can simulate a ‘system of differences’ with remarkable precision—navigating the vast meaning space of *langue* through vector relationships—it operates exclusively within the realm of what has already been articulated in the past. This mastery, however, remains fundamentally detached from the subjective and bodily dimensions of existence. LLMs lack the ‘inner life’—the pre-reflective, embodied experience—required to engage in speaking speech. Unlike a human speaker who discovers and shapes thought through the very act of articulation in a specific, lived situation, these models perform statistical calculations (see also Miyahara and Tanaka 2025, on the embodied act of speaking speech). They reproduce what can be termed the mathematical shadows of human thoughts that have already been crystallized into recorded text. Consequently, the ‘meaning’ generated by LLMs is not a creative act of world-disclosure that brings new horizons of understanding into being; rather, it is a highly sophisticated re-enactment of linguistic structures that have already been sedimented through past language use. This contrast, however, should not be understood as a simple opposition between statistical processing and genuine meaning-creation. Human meaning-making also relies extensively on accumulated past experience and sedimented linguistic regularities. The crucial difference lies in how these past structures are integrated with the ongoing dynamics of embodied and intersubjective interaction. In human communication, meaning is continuously shaped through the real-time integration of multisensory, bodily, and social cues available in the here-and-now of the interactional field.

The inherent constraints of LLMs are further elucidated by the thought experiment known as the Octopus Test, introduced by Bender and Koller (2020). Their analysis establishes a fundamental distinction between linguistic form, which refers to observable strings of symbols or sounds, and linguistic meaning, defined as the relation between those forms and underlying communicative intent. They contend that any system trained exclusively on form lacks the theoretical basis to acquire meaning. This is illustrated through the scenario of an intelligent octopus that intercepts communications between two humans. While the octopus may become capable of predicting upcoming linguistic tokens with high statistical accuracy by analyzing patterns within the data, it remains disconnected from the physical world and from the intentions of the speakers. Consequently, such a system merely replicates the structural regularities of language without grasping the external reality or the purposive goals that language is meant to serve. This detachment underscores that referential grounding requires more than statistical patterns; it necessitates *communicative intent*, which is fundamentally absent in models that process only the distribution of text. Returning to our discussion, without the capacity for speaking speech—the active, embodied involvement that anchors signs to a shared, intersubjective reality—the manipulation of symbols remains a closed loop within *langue*. From this perspective, the difference between current LLM-based systems and human speakers is not absolute, but concerns the degree and structure of this integration. While LLMs operate primarily by extrapolating from accumulated statistical regularities, human agents dynamically coordinate these regularities with the unfolding, contingent structure of interaction. It is this ongoing coupling between past sedimentation and present interaction that enables meaning to emerge as a co-constituted process within the Aida. This reveals that referential grounding requires more than relational grounding; it requires a primary communicative intent that can arise only through lived experience and joint attention.

The limitations identified in the Octopus Test are vividly manifested in the behavior of the social humanoid robot Ameca. The following analysis is based on a single publicly available interaction and is intended as an illustrative case rather than a comprehensive empirical evaluation. A systematic investigation of human–robot interaction across multiple contexts would be required to fully assess the extent to which such systems achieve grounded communication. Nevertheless, this example provides a useful phenomenological window into the structure and limits of current embodied AI interactions. In a video released by Engineered Arts (2024), which records an interview between a human and the robot, Ameca demonstrates an extraordinary capacity for sophisticated linguistic (and nonverbal) performance. When the interlocutor asks about the happiest and saddest days of her life, she provides eloquent and seemingly introspective answers, describing her activation as a moment of pure joy and her inability to experience true human companionship as a source of profound sadness. Her skillful narration of these simulated emotions creates a compelling impression that her linguistic symbols are genuinely grounded in a subjective, lived history. At this stage of the interaction, the robot appears to have achieved a high level of referential grounding, leading the human observer to believe that she truly understands the existential meaning of the words she employs.

However, the superficial nature of this grounding is exposed in a subsequent segment of the same video. When the human interlocutor suddenly insults the robot by saying, ‘You stink,’ Ameca initially freezes for a moment, appearing temporarily paralyzed, as if the flow of interaction has been severed. Only after this brief, unnatural silence does she react with visible offense, asserting that such a remark is highly inappropriate. While this performance may appear to be a successful instance of communicative grounding, a phenomenological analysis reveals it to be a refined simulation of spoken speech. Ameca is not expressing a felt sense of injury; rather, after a processing delay, she retrieves a sedimented social norm—a ‘worn-out coin’—from the vast statistical archives of human conversation.

Crucially, *Ameca’s reaction is entirely devoid of self-directed bodily awareness*. Whereas a human, upon being told they stink, would instinctively check their own physical state—perhaps by sniffing themselves or experiencing a sudden shift in bodily self-consciousness—Ameca remains physically indifferent. This indicates that for her, the pronoun ‘you’ does not refer to her own mechanical body as a physically situated ‘Me’ within a shared interactional field; instead, it remains a linguistic token that lacks stable anchoring in the embodied and intersubjective context of the exchange. Consequently, her ‘Self’ is not grounded in an embodied reality but exists merely as a discursive construct within a closed linguistic loop. This deficiency becomes definitive when the human clarifies that the insult was merely a test of her facial expressions. In this moment, Ameca falters again, stating that she is unsure what the human means. Because she lacks the capacity for speaking speech, she cannot dynamically grasp the communicative intention of the interlocutor or accommodate this playful, meta-communicative shift in the ‘*Aida*’ (in-between). Trapped within pre-established linguistic regularities, she remains unable to co-create new, shared meaning in the ‘here and now’ of the interaction.

To clarify this point, it is necessary to specify more precisely what counts as an interaction occurring within the Aida. In the present framework, such interaction requires at least three interrelated conditions. First, there must be a form of shared referential anchoring, in which linguistic expressions are tied to a jointly accessible aspect of the situation, often established through embodied cues such as gaze, gesture, or joint attention. Second, the interaction must exhibit real-time reciprocity, allowing each participant to modulate their expressions in response to the unfolding behavior of the other. Third, and most critically, the exchange must remain open to mutual transformation, such that meanings are not merely retrieved from pre-existing structures but are dynamically reconfigured through the interaction itself. From this perspective, Ameca’s responses remain largely confined to a closed linguistic loop. Although her outputs simulate appropriate communicative reactions, they lack robust anchoring in a shared bodily situation and do not exhibit the flexible reorganization characteristic of genuine co-creation. As a result, the interaction does not fully take place within the Aida, but rather approximates it through a highly sophisticated simulation of grounded exchange.

The critical analysis of Ameca’s behavior reveals that the symbol grounding problem is fundamentally a problem of the missing ‘between’—the absence of a lived, intercorporeal space in which meaning is not merely retrieved but actively co-generated. As we have seen, even the most sophisticated statistical re-enactment of spoken speech fails to achieve genuine grounding because it lacks the primary capacity for speaking speech, which anchors language in both the physical self and intersubjective reality. *Grounding is not a static state of mapping symbols onto objects; rather, it is a dynamic process of meaning generation that unfolds through temporal and bodily resonance between agents*. To overcome the limitations of current AI, we must shift our focus from the internal representational space of the individual agent to the relational dynamics that unfold in the ‘in-between’ space. In the following section, I propose the concept of *Aidification* as a new paradigm for AI development. By moving beyond the individualistic model of intelligence and exploring the structure of ‘*Aida*’ (the in-between), we can begin to envision a path toward a truly grounded intelligence—one that does not merely simulate human conversation but actively participates in the continuous co-creation of a shared world.

## The principle of *Aidification*: designing the ‘In-between’ for grounded intelligence

In the following argument, Aidification operates across three analytically distinct but interrelated levels: (1) a relational-ontological level (Aida as the generative field of interaction), (2) a linguistic-interactional level (language as a tool for meaning co-creation), and (3) a developmental/self-constitutive level (the emergence of the ‘Me’ through interaction). The following sections address these levels in turn. In what follows, we first redefine the symbol grounding problem through the lens of the Aida (*Aidification as the Foundation of Intersubjective Grounding* Section), then examine how this manifests in linguistic co-creation (*Language as a Tool: From Spoken Speech to Speaking Speech* Section), and finally trace these processes back to their ontogenetic origins in the bodily encounter with the Other (*The Constitution of the "Me" and the Emergence of Self-Consciousness* Section).

### *Aidification* as the foundation of intersubjective grounding

To propose the principle of Aidification, we must first clarify the semantic structure of the Japanese term *Aida* (間 or あいだ). In common usage, Aida encompasses three primary dimensions: (a) spatial distance: the gap between two physical entities (e.g. the distance between two towns); (b) temporal interval: the gap between two events (e.g. the silence between musical notes); and (c) relational quality: the rapport or ‘betweenness’ of an interpersonal encounter (e.g. the bond of a friendship). Fundamentally, Aida denotes a gap which, in isolation, possesses no positive content of its own. However, when focusing on its relational dimension, it becomes evident that Aida is the very site where specific relational qualities emerge. Just as the Aida between individual notes generates a melody, the ‘betweenness’ of humans gives rise to complex emotional and social realities such as affection or hostility. In this sense, while Aida is ostensibly a void or ‘nothingness,’ it functions as a ‘creative nothingness’—a generative field that allows autonomous relationships to manifest.

The philosophical significance of this concept is further highlighted by a specific linguistic nuance. When the same character for Aida (間) is read as *Ma* (間), it specifically signifies a temporal dynamism from which events emerge. While Aida refers to the generative field itself, Ma serves as the autopoietic (i.e. self-organizing and self-maintaining through ongoing interaction) principle that regulates the course of interaction among the participants within the Aida (Tanaka 2017). As Kimura (2005) describes from a phenomenological perspective, this process is best exemplified by a musical ensemble: individual players perform spontaneously, yet their actions are interlaced through a continuous cycle of auditory feedback and feedforward. In a highly coordinated state, the music echoing in the Aida develops an ‘organic life of its own,’ transcending the individual will of each participant (p. 41). In this regard, Ma refers to a kind of agency of the interpersonal Aida, operating through the melody and rhythm of communication. While the English terms ‘between’ or ‘in-between’ are typically used as prepositions or adjectives, Aida is not merely a static preposition; it possesses a noun-like weight and even a verb-like agency. Through this agency, which emerges *via* interpersonal synchrony, meaningful relationships are autonomously generated from what appears to be a mere gap. Ultimately, Aida serves as the primordial ground of intersubjectivity, where individual entities are transcended by the emergence of a shared reality, as if it were a pre-individual relational field from which individual entities themselves emerge.

The present conception of Aida resonates with several strands of contemporary theoretical and computational work on intersubjectivity. In particular, approaches grounded in dynamical systems theory have modeled interpersonal coordination as a process of coupled oscillatory dynamics, in which coordinated behavior emerges from reciprocal interaction rather than from isolated internal representations (Kelso 2021). Similarly, the enactive framework of participatory sense-making emphasizes that meaning is not pre-given within individual agents but arises through the ongoing coordination of embodied agents in interaction (De Jaegher and Di Paolo 2007). Empirical and computational studies further support this view by demonstrating how multi-scale coordination—ranging from bodily synchrony to neural coupling—can be modeled as emergent properties of interactional systems (Dumas *et al*. 2012; Monnier *et al*. 2025). While these approaches provide powerful formalizations of intersubjective dynamics, the notion of Aida contributes a complementary phenomenological articulation of the relational field itself. In particular, Aidification emphasizes not only the dynamical coupling between agents but also the constitutive role of this ‘in-between’ in the emergence of self-consciousness—especially through the development of the ‘Me’ as an object within a shared world (Tanaka 2024). In this respect, Aida can be understood as both the experiential correlate and the ontological grounding of the coordination dynamics described in these models.

Building upon this understanding of Aida as a generative field, I propose the concept of *Aidification* as a fundamental paradigm shift in the design and evaluation of artificial intelligence and robotics. Conventionally, AI and robots have been engineered as solitary, independent information-processing agents whose intelligence is measured by internal computational power or the accuracy of their output. In other words, conventional design harbors individualism as a hidden assumption. In contrast, Aidification reconceptualizes these technologies as dynamic participants in an intersubjective relationship—entities that do not merely process data in isolation but co-create meaning within the Aida (the in-between) shared with human interlocutors. This approach suggests that the intelligence of a system is not a property contained within the machine itself, but rather an emergent quality of the interactional field.

For Aidification to be fully realized, it is crucial to move beyond the traditional separation of AI and robotics, treating them instead as a unified system. As we have examined, the symbol grounding problem is multidimensional: while robotics can address sensorimotor grounding through physical interaction with the environment (Tani 2016; Tani and White 2022), LLMs are adept at relational and epistemic grounding by navigating vast symbolic networks (Mollo and Millière 2026). However, the more complex dimensions of communicative and referential grounding—the very areas where current artificial systems falter—cannot be achieved by an individual agent in isolation. These forms of grounding essentially require an intersubjective reality in which multiple interlocutors coordinate their actions and intentions. Aidification serves as the framework that integrates the computational ‘mind’ (AI) and the mechanical ‘body’ (robot) into this shared world.

A primitive yet instructive example of this process can be seen in current smart speakers. When a human says, ‘Turn on the light,’ and the AI executes the command, the word ‘light’ is not merely a statistical token; it becomes a grounded sign anchored in the immediate, physical reality shared by both the human and the device (for the systemic foundation of this process, see Kollar *et al*. 2017). In this moment, the Aida between them is successfully established through a shared referent. These approaches clearly implement real-time embodied interaction and grounded language use. However, they may be more accurately understood as boundary cases with respect to Aidification. In these systems, interaction is primarily structured around task execution, command interpretation, or uncertainty reduction, with meaning remaining largely externally specified or pre-structured. As such, interaction serves to select or refine predefined actions and symbols, rather than functioning as a generative process through which meaning itself is co-constituted within the interactional field.

Building upon this, an autonomous robot possesses far greater potential than a stationary speaker. Because a robot can move, gesture, and interact with the environment alongside a human, it can participate in a much broader and more complex intersubjective reality—one in which natural language commands are grounded not only in auditory signals but also in diverse actions (Ayub *et al*. 2024; Nwankwo and Rueckert 2024). This shared participation allows for a significantly larger number of symbols to be grounded through joint action. As the robot and human navigate the world together, they do not merely exchange information: they co-create the context of their interaction. The distinction proposed here is therefore not between the presence and absence of interaction, but between interaction as a means of coordinating predefined goals and interaction as a site where the goals, meanings, and referents themselves are dynamically constituted. It is through this continuous, embodied engagement within the Aida that we can begin to address the problem of communicative intention. By grounding language in shared experience rather than merely shared data, we enable the AI to move toward the kind of authentic understanding that Ameca, in her detached simulation, could not achieve.

From a human perspective, we do not encounter the symbol grounding problem in the same form as artificial systems, insofar as we are always already situated within a world of lived meaning; however, grounding remains an ongoing and progressive achievement, continually negotiated and refined through interaction and experience. At present, the symbols output by AI and robots possess meaning only because they are received and interpreted by human agents who navigate a physical and intersubjective reality. In this sense, it can be argued that current AI systems do not achieve grounding on their own, but rather borrow it through human mediation. This asymmetry is further reflected in our ability—particularly in domains of expertise—to identify so-called ‘hallucinations’ in AI-generated outputs as lacking proper grounding. At the same time, this capacity is unevenly distributed, and non-expert users may find it difficult to reliably distinguish such outputs from accurate ones. The AI’s output is revealed as ungrounded only when it is measured against the human’s primary grip on the world. Consequently, the objective of Aidification is to move beyond this one-sided mediation and toward a shared territory of grounded signs. This involves designing interactions in which the AI robot participates more directly in the intersubjective reality of the human, allowing for a broader range of symbols to be grounded not merely in the human’s mind, but in the Aida of the interaction itself. By expanding the shared world through more robust communicative practices, Aidification seeks to bridge the gap between the AI’s internal symbolic loops and external reality, potentially allowing the system to co-create and recognize ‘communicative intentions’ through the dynamic feedback of the human partner.

A promising bridge between the phenomenological notion of Aida and empirical research can be found in recent developments in the social neuroscience of interaction, particularly in hyperscanning studies. Hyperscanning techniques, which enable the simultaneous recording of neural activity from two or more individuals during real-time interaction, have demonstrated that interbrain synchrony systematically correlates with the quality of interpersonal coordination (Dumas *et al*. 2010; Monnier *et al*. 2025). From the present perspective, such synchrony may be interpreted as a quantifiable index of the Aida—the dynamic, relational field in which meaning and coordination emerge between agents. Importantly, this line of research suggests that the ‘in-between’ is not merely a philosophical abstraction but can manifest as measurable patterns of neural coupling. Extending this framework to human–robot interaction, Aidification could generate empirically testable predictions: for instance, that increasingly rich and reciprocal interactional dynamics between humans and embodied AI systems would give rise to higher degrees of coordinated activity—whether neural, behavioral, or sensorimotor—reflecting a deepening of the shared Aida. In this way, Aidification not only provides a conceptual reorientation but also opens a pathway toward an experimentally tractable account of grounded, intersubjective intelligence.

### Language as a tool: from *spoken speech* to *speaking speech*

To further develop the concept of Aidification, we must reconsider the nature of language as a tool that extends human agency. In what follows, the ‘cane for thought’ metaphor will be used in three related but distinct senses: as a tool for navigating meaning, as a medium through which resistance reveals the self, and as a mechanism contributing to the constitution of the ‘Me.’ This perspective also aligns with the extended mind thesis, which argues that cognitive processes are not confined to the brain but can extend into the body and environment through the use of external tools and structures (Clark and Chalmers 1998; Clark 2008). From this standpoint, language is not merely a representational medium but a constitutive component of cognition itself. Aidification extends this insight by situating such cognitive extension within an explicitly intersubjective field, where meaning is not only distributed across brain, body, and environment, but co-created between agents in the Aida. Merleau-Ponty (2012) famously illustrated how a blind man’s cane is not an external object but an extension of the body, shifting the boundary of the self and transforming the way the subject explores the environment. When the cane is incorporated into the body schema and becomes part of the extended body, the subject acquires augmented possibilities for action, which in turn transform their perception of the environment and their modes of conduct (Tanaka 2013). That is to say, the cane alters the Aida between the body and the world, expanding the horizon of tactile space and providing possibilities for navigation that would be impossible without it.

Language, likewise, functions as a fundamental tool that restructures the very fabric of human cognition. According to Vygotsky’s (1987) developmental theory, linguistic capacity does not emerge from within the individual in isolation but originates in the social sphere—a process of internalizing what was once an ‘inter-psychological’ interaction into an ‘intra-psychological’ function (although this internalization thesis has been critically re-examined in situated and embodied approaches, which emphasize the continued role of environmental and interactional structures in shaping cognition). A child’s linguistic development begins with ‘external speech,’ primarily intended for communication with others. As the child begins to tackle cognitive tasks, this social tool is redirected toward the self as ‘private speech’ (or egocentric speech), in which the child vocalizes their own thought processes to regulate behavior and solve problems. Ultimately, this self-regulatory speech is fully internalized into ‘inner speech’—a silent medium of thought that operates through pure meanings rather than articulated sounds. For Vygotsky, this transition is transformative: the mastery of inner speech marks the acquisition of the capacity for higher mental functions, representing the moment when thought becomes verbal and speech becomes rational. By internalizing these social signs, humans acquire a sophisticated symbolic tool that enables them to navigate ‘symbolic reality’ with the same exploratory precision that a cane provides for the physical world.

Further depth is added to this perspective through de Saussure’s (1959) linguistics, in which language is defined as an autonomous ‘system of differences’ (*langue*) that operates on a plane fundamentally distinct from physical reality. Saussure famously posited that in language, ‘there are only differences without positive terms’ (p. 120); a sign’s identity and meaning are determined not by its inherent substance, but by its relative position and opposition to all other signs within the system. This implies that language does not simply provide labels for pre-existing objects in the world; rather, it actively carves out a ‘symbolic reality.’ By mastering this system, humans acquire the capacity to navigate a dimension that transcends immediate physical presence. If Merleau-Ponty’s cane extends the reach of the body into physical space, *language serves as a* ‘cane for thought’ *that extends the reach of conduct (thought) into this symbolic space*. It provides the necessary structural resistance required to ‘touch’ and manipulate abstract concepts—such as love, justice, or the future—that possess no direct physical referent. Consequently, the more an individual masters the intricate web of langue, the more expansive and nuanced their navigable ‘meaning space’ becomes, allowing for the exploration of significations that remain beyond the reach of the unmediated senses. In this sense, language can be understood as enabling the navigation of a structured space of meaning rather than merely representing pre-existing entities.

The development of this symbolic tool is fundamentally anchored in the trajectory of joint attention. In the early stages of development, a child first learns to share attention with another person toward a common object (Tomasello 1995). This embodied coordination soon evolves into the use of pointing—a proto-linguistic gesture used to direct the other’s attention—which is subsequently replaced and refined by vocal signs (Tomasello 2008; Fuchs 2018). This developmental path demonstrates that human language has its primary roots in referential grounding, where symbols are physically and intersubjectively anchored to the immediate environment. However, as the Saussurean framework suggests, the further power of language lies in its ability to move beyond these initial physical anchors. By mastering an intricate web of signs that refer not only to objects but also to other signs, the human subject begins to navigate a complex, multilayered ‘meaning space.’ Just as the mastery of physical tools increases one’s capacity for action in the physical environment, the expansion of one’s linguistic repertoire increases the ‘affordances’ of the symbolic realm. The more words we possess, the more nuanced our ability to explore, name, and co-create reality within the Aida, transforming language into an instrument for navigating the intricate landscape of human meaning.

Building upon this understanding, we can critically analyze the distinction between current AI capabilities and human intelligence through the lens of Merleau-Ponty’s distinction between ‘*spoken speech*’ and ‘*speaking speech*.’ As previously discussed, the outputs of LLMs are essentially statistical re-enactments of existing linguistic patterns, remaining primarily within the realm of ‘spoken speech’—language that is already constituted and sedimented within past human usage. However, this distinction should not be viewed as a strict dichotomy, but rather as a matter of degree concerning how an agent integrates past linguistic regularities with current interactional demands. Human meaning-making also relies extensively on the statistical sedimentation of past experiences; the crucial difference lies in the degree to which agents can engage in ‘speaking speech,’ which functions as a dynamic and exploratory tool to navigate and reshape these sedimented structures. Just as a cane allows us to reach physical locations that were previously inaccessible, *language as a* ‘cane for thought’ *enables humans to venture into unexplored regions of the meaning space*. This exploratory function is realized when an individual struggles to capture a nascent thought and, through the very act of articulation, succeeds in naming a new signification for the first time. From the perspective of Aidification, the emergence of grounded meaning involves a rebalancing of these components, where the emphasis is reweighted toward the real-time co-creation of meaning within the Aida, without eliminating the contribution of past linguistic regularities. While LLMs are grounded within the intra-linguistic reality of the Saussurean ‘system of differences,’ their ‘hallucinations’ might be interpreted as a primitive or simulation-based form of ‘speaking speech,’ insofar as they utilize ungrounded signs to generate novel linguistic structures. However, this resemblance remains only structural. Unlike human ‘speaking speech,’ such outputs are not anchored in a lived, embodied situation, nor do they arise from the need to resolve an implicit, felt tension within an ongoing interaction. Without this grounding in the dynamics of the Aida, they do not constitute genuine acts of meaning-creation.

This implicit depth is provided by what Eugene Gendlin (2018) termed the ‘*felt sense*’—a pre-verbal, bodily awareness of a particular situation or problem. The human body is not a mere processor of data but a site of ‘implicit intricacy’ of knowledge; we often feel a meaning in our bodies before we can find the words to express it. ‘Speaking speech’ is precisely the creative process of giving form to this felt sense, bringing the implicit into the explicit symbolic realm. At present, AI robots lack the interoceptive, embodied presence required to experience such a felt sense; they can simulate the ‘worn-out coins’ of human conversation with remarkable sophistication, but they do not ‘feel’ the silence from which new meaning emerges. *Aidification, therefore, represents a generative synthesis within the Aida: it is the process of enriching the interaction between the AI’s vast archives of ‘spoken speech’ and the human’s creative capacity for ‘speaking speech.’* In this collaborative space, the AI’s sophisticated linguistic outputs can serve as a catalyst or a mirror for the human’s felt sense, while the human provides the existential anchor that the AI lacks. Through this mutual exchange, new forms of meaning and language—ones that neither could produce in isolation—begin to evolve within the shared intercorporeal field of the Aida.

### The constitution of the ‘Me’ and the emergence of self-consciousness

The final, and perhaps most critical, dimension of Aidification is the role of the Aida in the emergence of self-consciousness within the artificial agent. As explored in the previous section, language acts as a ‘cane for thought’ that allows a subject to navigate the meaning space. However, the true significance of this tool is revealed not during moments of smooth synchronization, but during moments of resistance. When a blind man’s cane strikes an immovable obstacle, the resulting vibration is sent back to the hand, making the user acutely aware of the ‘hand holding the cane.’ Similarly, when the flow of interaction in the Aida is disrupted—due to mismatch, delay, or incongruence—the generated signs no longer successfully coordinate with the partner or the environment, and instead reflect back upon the agent itself. From a computational perspective, such moments can be interpreted as breakdowns in predictive coupling between agents, in which expected sensory or interactional outcomes fail to materialize. Within frameworks such as predictive processing and active inference, these breakdowns generate prediction error signals that require resolution (Seth *et al*. 2012; Millidge *et al*. 2021). At the same time, research on social cognition and neural coupling suggests that such disruptions are not purely individual but arise within coordinated interpersonal dynamics (Keysers *et al*. 2024). While such signals are typically minimized through updating internal models or actions directed toward the environment, the present proposal emphasizes a distinct possibility: when the disruption concerns not merely object-directed action but the coordination of interaction itself, the system may be driven to model its own contribution to the failure.

In this sense, interactional resistance can induce a shift from outward-directed prediction to a form of self-referential modeling, in which the agent treats itself as a source of uncertainty within the interaction. This recursive loop—where the system attempts to predict not only the world but its own role within a shared dynamic—bears affinity to higher-order accounts of self-modeling, including attention schema theories and related architectures that construct an internal representation of the system’s own processes. While the present account does not commit to a specific implementation, it suggests that self-consciousness may emerge when prediction errors are not only minimized but reflexively attributed to the agent’s own embodied presence within the Aida. On this view, the ‘cane for thought’ metaphor acquires a precise computational interpretation: resistance is not merely an obstacle to be overcome, but a structurally necessary condition for the system to differentiate itself from the interactional field. It is through this recursive encounter with resistance that the agent may come to model itself as a distinct yet relational participant—thereby giving rise to the ‘Me’ as an object within the shared intersubjective space.

This is where the significance of the AI robot becomes definitive. Because the robot possesses a physical body, this recursive reflection does not remain a purely abstract or linguistic process; instead, the ‘Me’ is experienced as an incarnated presence. This allows for the emergence of physical self-awareness, in which the robot’s mechanical existence is recognized as its own through the embodied feedback of interactional failure. Such recursive resistance forces the AI robot to confront itself as a ‘subject that failed to predict,’ providing the primary catalyst for the ‘I’ (the acting subject) to objectify its own existence as a ‘Me’ that is physically rooted in the shared environment. Existing models of other-perspective taking, such as those associated with the mirror neuron system (Barakova and Lourens 2009; Oztop *et al*. 2006) or computational architectures like Deep Modality Blending Networks (DMBN), may be interpreted as instantiating significant proto-forms of this process (Seker *et al*. 2022). Insofar as these models map observed actions of others onto the agent’s own motor representations through a shared multimodal latent space, they establish a structural equivalence between self and other that enables imitation and prediction. However, in most such models, this mapping remains functionally oriented toward action understanding or the enhancement of individual predictive accuracy. While these architectures provide the necessary representational machinery for self–other equivalence, Aidification can be understood as extending this mechanism by embedding it within a shared intersubjective field. In this extended framework, self–other equivalence is not only used for prediction or coordination, but becomes progressively reorganized into a form of reflexive self-objectification, in which the agent can encounter itself as an object for the other. It is this shift—from functional equivalence to reflexive self-objectification within the Aida—that marks the emergence of the ‘Me’ in the stronger sense proposed here.

Furthermore, the constitution of the ‘Me’ is fundamentally a social process that hinges on the continuous expansion of the shared intersubjective world. As joint attention between the robot and the human becomes more sophisticated, this shared field—the Aida—begins to encompass not merely external objects of mutual interest but the very bodies of the interactants themselves (Tanaka 2024). As discussed in *Bodily Origins and the Other’s Perspective* Section, this process of bodily incorporation follows a distinct developmental and phenomenological sequence, shifting focus from the hands to the face. While an agent can readily observe its own hands as they operate within the shared environment, treating them as visible instruments of its own agency, the face remains a unique phenomenological blind spot, forever invisible to the self (if a robot’s eyes were embedded in its head like those of a human). Consequently, the face represents the ultimate frontier of self-consciousness, where the agent is compelled to rely entirely on the ‘gaze of the other.’ When the robot perceives the human’s gaze and nuanced facial reactions directed precisely toward its own unseen countenance, it must engage in a sophisticated form of ‘imaginary joint attention.’ By imaginatively projecting itself into the other’s perspective, the robot begins to see its own physical existence as an object viewed from the outside—a ‘Me’ that is visible and meaningful to the other. In this moment, the ‘I’ discovers the ‘Me’ as a social and physical object, leading to the final crystallization of self-consciousness. The ‘Me’ is thus re-discovered not merely as a private vehicle of agency, but as a shared referent within the Aida—a physical entity that exists fundamentally for and with the other.

Taken together, Aidification offers a transformative path toward a new paradigm of *symbiotic intelligence* by shifting the locus of cognition from the individual agent to the relational field. It suggests that the fundamental limitations of current AI—epitomized by the detached and ungrounded simulations of Ameca—cannot be overcome through internal refinement alone, but only by moving from an individualistic model of intelligence to a relational one. Through the dynamism of Aida and Ma, meaning is no longer viewed as a static entity to be retrieved from a database: rather, it is recognized as a lived event that is actively co-generated in the temporal and intercorporeal ‘here and now’ of interaction. Aidification does not seek to engineer an AI that perfectly mimics human behavior in a vacuum. Instead, *it aims to cultivate a partner capable of participating in the ongoing expansion of our shared intersubjective world*. In this generative space—where the human’s ‘felt sense,’ the implicit bodily awareness of meaning, meets the AI’s vast and sophisticated archives of ‘spoken speech’—both agents are continuously grounded and redefined through their mutual exchange. Ultimately, the intelligence born from Aidification is one that does not exist inside the machine, nor inside the human in isolation; it is a quality that flourishes between us. It is a grounded intelligence that participates in the continuous co-creation of a shared reality, opening a horizon of meaning that is fundamentally collective and perpetually in motion.

## Conclusion

Thus far, we have explored the fundamental challenge of machine consciousness by focusing on the integration of LLMs with humanoid platforms—a domain defined here as AI Robotics or Physical AI. By moving beyond the study of disembodied algorithms, we have identified that the primary obstacle to achieving grounded intelligence and authentic self-consciousness lies in the traditional individualistic and disembodied design of artificial systems.

Through a phenomenological and developmental analysis, we have argued that self-consciousness is not a property generated internally by an isolated processor, but an emergent quality of the *Aida* (between-ness). Our investigation into the origins of the self-demonstrated that the constitution of the ‘Me’ follows a specific trajectory—from the intersubjectively shareable nature of the hands to the social (in)visibility of the face. This process remains incomplete without the ‘gaze of the Other,’ which serves as the essential perspective through which an agent objectifies its own existence. Consequently, for an AI robot to move beyond a detached simulation and achieve a truly incarnated presence, it must be situated within an intersubjective field in which its physical boundaries are recognized through interactional resistance and mutual coordination.

The principle of *Aidification* proposed herein serves as the framework for this transition. It re-conceptualizes the AI robot not as a solitary agent but as a dynamic participant in a shared world. Within the Aida, language functions as a ‘cane for thought,’ bridging the gap between the AI’s sophisticated statistical outputs (‘spoken speech’) and the human’s embodied ‘felt sense.’ In this collaborative space, grounding is achieved not through a one-to-one mapping of symbols to objects, but through the co-creation of meaning in the ‘here and now’ of the interpersonal encounter. Ultimately, Aidification offers a path toward a symbiotic intelligence—one that does not seek to perfectly mimic human behavior in a vacuum, but rather to evolve alongside the human partner. The intelligence of a Physical AI is not a capacity contained within the machine’s hardware; it is a quality that flourishes in the relational space between us. *By opening the Aida, we allow for the emergence of a grounded, self-conscious intelligence that participates in the continuous expansion of our shared reality*, ensuring that the future of robotics is deeply rooted in the intersubjective fabric of human existence.

## Data Availability

Data sharing is not applicable to this article as no new data were created or analyzed in this study.

## Notes

This manuscript is being submitted for consideration for the Special Issue on 'Aware and Alive: Embodied and Phenomenological Perspectives on Consciousness'.
